# P-1272. Hepatitis A Investigation and Onsite Response in a Retirement Facility, New York City, 2023

**DOI:** 10.1093/ofid/ofae631.1453

**Published:** 2025-01-29

**Authors:** Brittan P Badenhop, HaeNa Waechter, Roisin McElroy, Julia Latash, Tingting Gu-Templin, Judy Chen, Mike O Antwi, Faina Stavinsky, Rameez Barnes, Bindy Crouch, Tristan D McPherson

**Affiliations:** New York City Department of Health and Mental Hygiene, Queens, New York; New York City Department of Health and Mental Hygiene, Queens, New York; New York City Department of Health and Mental Hygiene, Queens, New York; New York City Department of Health and Mental Hygiene, Queens, New York; New York City Department of Health and Mental Hygiene, Queens, New York; New York City Department of Health and Mental Hygiene, Queens, New York; New York City Department of Health and Mental Hygiene, Queens, New York; New York City Department of Health and Mental Hygiene, Queens, New York; New York City Department of Health and Mental Hygiene, Queens, New York; New York City Department of Health and Mental Hygiene, Queens, New York; New York City Department of Health and Mental Hygiene, Queens, New York

## Abstract

**Background:**

Hepatitis A is a contagious liver infection that can be transmitted by contaminated food consumption. On August 13, 2023, a healthcare provider notified the New York City Health Department of a hepatitis A case in a food handler who worked at a retirement facility while infectious. Medical frailty, mobility, and transportation limitations complicated exposed persons’ access to postexposure prophylaxis (PEP) within the recommended 2-week window. During the COVID-19 pandemic, the Health Department developed capacity to provide onsite SARS-CoV-2 testing and vaccination services within congregate settings to prevent and respond to outbreaks. We used this infrastructure to deliver timely PEP to contacts and prevent an outbreak.

**Methods:**

Using a risk-assessment algorithm, we evaluated the food handler’s hand hygiene, type and quantity of food prepared, and contact with ready-to-eat food. Anyone who ate food the worker prepared while infectious, defined as 2 weeks before symptom onset, was considered exposed. Following Advisory Committee on Immunization Practices guidance, 1 dose of single-antigen hepatitis A vaccine was advised for exposed adults, with coadministration of 0.1 mL/kg human immunoglobulin (IG) for those age > 40 years. Food handlers were required to receive PEP to prevent workplace exclusion. To enable timely PEP receipt, the Health Department worked with facility staff and a vendor to host an onsite clinic on August 23, 2023.

**Results:**

We identified 84 exposed persons, including 42 (50%) residents, 10 (12%) food handlers, and 32 (38%) staff. Median age of those exposed was 74 years (interquartile range [IQR]: 58–88 years); residents’ median age was 89 years (IQR: 84–91 years). Of 84 exposed persons, 39 (46%) received PEP; 30 (77%) received vaccine and IG, 1 (3%) IG only, and 8 (21%) vaccine only. Seven exposed persons who received only vaccine were eligible for IG. All 10 (100%) exposed food handlers, 18 (43%) residents, and 11 (34%) staff received PEP. One secondary case was identified in a resident who declined PEP.

**Conclusion:**

This intervention ensured residents were protected and prevented work exclusions. Providing timely, accessible clinical services within congregate settings, as was commonly done for COVID-19, might effectively prevent the spread of other infections.

**Disclosures:**

**All Authors**: No reported disclosures

